# Selection and Validation of Novel RT-qPCR Reference Genes under Hormonal Stimuli and in Different Tissues of *Santalum album*

**DOI:** 10.1038/s41598-018-35883-6

**Published:** 2018-11-30

**Authors:** Haifeng Yan, Yueya Zhang, Yuping Xiong, Qingwei Chen, Hanzhi Liang, Meiyun Niu, Beiyi Guo, Mingzhi Li, Xinhua Zhang, Yuan Li, Jaime A. Teixeira da Silva, Guohua Ma

**Affiliations:** 1 Guangdong Provincial Key Laboratory of Applied Botany, South China Botanical Garden, the Chinese Academy of Sciences, Guangzhou, 510650 China; 2 University of Chinese Academy of Sciences, Beijing, 100039 China; 3 P.O. Box 7, Miki-cho Post Office, Miki-cho, Ikenobe 3011-2, Kagawa-ken, 761-0799 Japan; 4 Genepioneer Biotechnologies Co. Ltd, Nanjing, 210014 China

## Abstract

Reverse transcription quantitative real-time polymerase chain reaction (RT-qPCR) is a widely used technique to investigate gene expression levels due to its high throughput, specificity, and sensitivity. An appropriate reference gene is essential for RT-qPCR analysis to obtain accurate and reliable results. To date, no reliable reference gene has been validated for the economically tropical tree, sandalwood (*Santalum album* L.). In this study, 13 candidate reference genes, including 12 novel putative reference genes selected from a large set of *S*. *album* transcriptome data, as well as the currently used β-actin gene (*ACT*), were validated in different tissues (stem, leaf, root and callus), as well as callus tissue under salicylic acid (SA), jasmonic acid methyl ester (MeJA), and gibberellin (GA) treatments using geNorm, NormFinder, BestKeeper, Delta Ct and comprehensive RefFinder algorithms. Several novel candidate reference genes were much more stable than the currently used traditional gene *ACT*. *ODD* paired with *Fbp1* for SA treatment, *CSA* and *Fbp3* for MeJA treatment, *PP2C* and *Fbp2* for GA treatment, as well as *Fbp1* combined with *Fbp2* for the total of three hormone treatments were the most accurate reference genes, respectively. *FAB1A*, when combined with *PP2C*, was identified as the most suitable reference gene combination for the four tissues tested, while the combination of *HLMt*, *PPR* and *FAB1A* were the most optimal reference genes for all of the experimental samples. In addition, to verify our results, the relative expression level of the *SaSSy* gene was evaluated by the validated reference genes and their combinations in the three *S*. *album* tissues and under MeJA treatment. The evaluated reference genes in this study will improve the accuracy of RT-qPCR analysis and will benefit *S*. *album* functional genomics studies in different tissues and under hormone stimuli in the future.

## Introduction

Reverse transcription quantitative real-time PCR (RT-qPCR) is a popular technique used to monitor the level of mRNA because of its high sensitivity, accuracy, specificity and efficiency^1^. To interpret the expression profiles of a target gene accurately and reliably, normalization of gene expression data using reference genes is essential in relative quantification analysis by RT-qPCR. Ideally, excellent reference genes should have a constantly stable or minimal variable expression in experimental conditions^2^. A few reference genes such as *18* *S rRNA* (18 S ribosomal RNA), *TUBA* (α-tubulin), *EF1A* (elongation factor 1α), *ACTB* (β-actin), and *GAPDH* (glyceraldehyde-3-phosphate dehydrogenase)^2^, which show relatively high levels of expression, are frequently used for RT-qPCR analysis in plants^3,4^. However, increasing evidence has demonstrated that the expression levels of these traditional reference genes vary considerably in different samples and under different experimental conditions^3,5,6^. Therefore, it is necessary to select and validate reference genes according to specific samples and experimental conditions.

*Santalum album* L., commonly known as sandalwood, is a hemiparasitic tropical tree distributed in India, Indonesia, Malaysia, and Australia^7^. It is famous for its valuable essential oil extracted from aromatic heartwood and roots that are used in aromatherapy, perfumes, cosmetics, medicine and sacred unguents^8,9^. A number of functional genes and their expression levels have been characterized and studied in *S*. *album* in recent years^7,10–13^. As far as we known, the traditional housekeeping gene *ACT* (β-actin) was the only reference gene used to date^10,12,14,15^, and there has been no systematic validation and evaluation of reference genes for RT-qPCR analysis in *S*. *album*.

In this study, 13 candidate reference genes, including 12 novel genes selected from a large set of RNA-seq data in three different tissues (stem, leaf, and root) of *S*. *album*, as well as the currently used traditional housekeeping gene *ACT*, were assessed by RT-qPCR. Five statistical algorithms (geNorm^16^, NormFinder^17^, BestKeeper^18^, Delta Ct^19^ and RefFinder^20^) were used to evaluate the expression stability of these putative reference genes. Furthermore, the key gene *SaSSy* for synthesizing the main component of sandal oil was investigated to validate the suitability of the newly identified stable reference genes. This work validated a set of more stable novel reference genes and will facilitate, expand and fortify gene expression analysis in different tissues and under hormone treatment of *S*. *album*.

## Results

### Selection of candidate reference genes based on transcriptome datasets

Based on the RNA-seq expression data previously published^12,13,15^, 12 genes (Table 1, Supplementary Table S1) with a CV ranging between 9.75% and 11.95% were selected. All 12 selected genes were newly identified candidate reference genes, as follows: contained a FYVE domain necessary for *FAB1* gene *FAB1A*, protein with unknown function (*UK*), F-box protein (*Fbp1*, *Fbp2* and *Fbp3*), cytochrome c biogenesis protein CCS1 (*CCS1*), pentatricopeptide repeat-containing protein (*PPR*), coatomer subunit alpha-1 (*CSA*), probable 2-oxoglutarate-dependent dioxygenase (*ODD*), probable protein phosphatase 2 C (*PP2C*), probable histone-lysine *N*-methyltransferase ATXR3 (*HLMt*), and 40 S ribosomal protein S8 (*S8*). The currently widely used housekeeping *ACT* gene, although not in the rank of most stable genes, was also assessed in our study for comparison.

**Table 1:** Selected candidate reference genes, primers, *T*_*m*_ and KS-test p values, and amplicon characteristics.

Gene name	Description	GeneBank accession number	Primer sequence (5′-3′) forward/reverse	*Tm* (^o^C)	Amplicon length (bp)	Amplicon efficiency (%)	R^2^	KS-test p value
*FAB1A*	With FYVE domain necessary for FAB1 gene	MG282422	AGCAGTTCTCAAAGGAGCTAAA	62	104	109.168	0.998	0.826
			ACCTTCGTGCGACAACTAAA					
*UK*	Function unknown protein	MG282423	TTTGGCAGTGATCGGTATCC	62	105	112.855	0.996	0.302
			CCTCTGTGTTAGGTAGCTTTGG					
*Fbp1*	F-box protein	MG282425	TGGCGTGTCCTGTTTCTATC	62	84	106.237	0.995	0.178
			CGCACTCCATAGGTTTCTTCT					
*CCS1*	Cytochrome c biogenesis protein CCS1	MG282427	GGCCCAATTGGATTTCTCTCTA	62	103	108.210	0.995	0.410
			GCAAACTTACTTCTCCGCTTTC					
*PPR*	Pentatricopeptide repeat-containing protein	MG282429	TGCTGAATAGTGCCGGTAAG	62	126	108.001	0.998	0.310
			TCTCCTTCATCTCATCCCAAATC					
*CSA*	Coatomer subunit alpha-1	MG282432	GCCAATATACCGAGGACAGAAG	62	104	106.05	0.999	0.505
			CAACCGCAAGATCACAAACAG					
*Fbp3*	F-box protein	MG282433	CCTCGTGTACTGGGAAATGG	62	110	107.471	0.998	0.243
			GCAAGAACGCAATGCCTAAA					
*ODD*	Probable 2-oxoglutarate-dependent dioxygenase	MG282424	TTTAGCATTGGGTGGGACTC	62	110	94.75	0.998	0.639
			CTTGGCGATTTGCATTGGTTA					
*PP2C*	Probable protein phosphatase 2C	MG282426	ACTGACCAGGCAATCCTTTC	62	92	93.919	0.994	0.391
			ATCCATAACCTTCGGCCATTTA					
*HLMt*	Probable histone-lysine N-methyltransferase ATXR3	MG282428	TGCTGAGGAAGACCAGGATA	62	110	105.477	0.998	0.242
			CACCAAGACCCTTCCGATAAG					
*Fbp2*	F-box protein	MG282430	CGAAGCCTGGTTCACTCTATG	62	94	98.121	0.997	0.051
			AAGCTAAGCCTCTGCAATGT					
*S8*	40 S ribosomal protein S8	MG282431	CCCGAGGATGATCTGGATAAC	62	88	110.782	0.995	0.266
			CATTACTGGTGAACCCAACAC					
*ACT*	actin	EF452617	GGATCCACGAGACTACCTACA	62	90	99.872	0.998	0.988
			GAGCCACACTGAGCACAATA					
*SaSSy*	SaSSy	JX826486.1	CCTTCCTGATCTTCTGCACTAC	62	91	105.997	0.993	_
			ATTATCGCCTCTTGCCATCTC					

### Primer specificity, amplification efficiency and expression profile of candidate reference genes

The specificity of primer pairs for each candidate reference gene was verified by 2% agarose gel electrophoresis with a single expected size product (Supplementary Fig. S1), and further demonstrated by melting curve analysis with a single peak (Supplementary Fig. S2). The cDNA-free template controls (ddH_2_O as template) showed no obvious melting curve products (data not shown). All these results confirmed the specificity of all primer pairs and the absence of DNA and other contaminating materials during RT-qPCR amplification.

The amplification efficiency for primer pairs of all candidate reference genes ranged from 93.919% (*PP2C*) to 112.855% (*UK*), and the *R*^2^ values lay between 0.994 (*PP2C*) and 0.999 (*CSA*) (Table 1).

**Figure 1:**
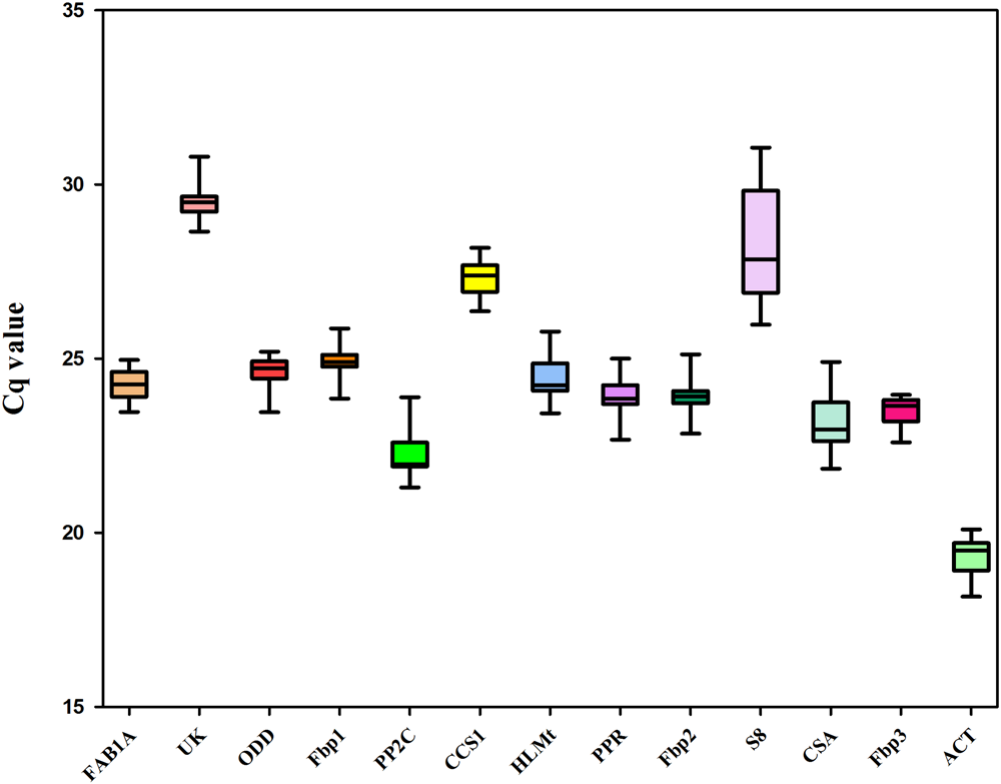
Distribution of Cq values of 13 candidate reference genes in all experimental samples. Boxplot graph showing maximum, minimum values, medians and 25/75 percentiles.

The expression profiles of the 13 candidate reference genes in all experimental samples was assessed by RT-qPCR using the Cq value for each sample, after testing for normality using the KS-test in which a p value > 0.05 was considered as normal. All KS-test p values of each sample were greater than the 0.05 cut-off value (Table 1), the average Cq values ranged from 19.8 to 28.96 (Fig. 1), and the majority of average Cq values lay between 23 and 25, which indicates that the expression of all candidate reference genes fitted within a suitable reference gene expression level (15 < Cq < 30)^21^. As shown in Fig. 1, the gene with highest expression was *ACT* (with the lowest Cq value), and the gene with the lowest expression was *UK* (with the highest Cq value). The candidate reference gene names, GeneBank accession numbers, primer sequences, *T*_*m*_ values, amplicon lengths, amplification efficiencies, *R*^2^ values, and KS-test p values are listed in Table 1.

### Expression stability of candidate reference genes in different tissues and under hormone treatment of *santalum album*

According to GeNorm analysis (Table 2), *M* values of all of the candidate reference genes tested were below 1.5, indicating that they all had relatively stable expression. Among all four tissues tested (Table 2), *FAB1A* and *PPR* were the most stable genes, while *Fbp1* was the least stable gene. For salicylic acid (SA) treatment (Table 2), *FAB1A* and *Fbp3* were the most stable reference genes. For jasmonic acid methyl ester (MeJA) (Table 2), *PP2C* and *CSA* were the top ranked genes. *PP2C* and *CCS1* ranked as the most stable reference genes for gibberellin (GA) treatment (Table 2). *PPR* and *Fbp2* (Table 2) were the most stable reference genes in all three hormone treatment sample sets. As for the total experimental samples (Table 2), *HLMt* and *PPR* were the most stable reference genes.

**Table 2:** Expression stability of 13 candidate reference genes calculated by GeNorm, NormFinder, BestKeeper, Delta Ct and RefFinder.

Group	Rank	GeNorm		NormFinder		BestKeeper		Delta Ct		RefFinder	
		Gene	MV	Gene	MV	Gene	CV ± SD	Gene	SV	Gene	SV
**Total tissues**	1	*FAB1A*	0.45	*PP2C*	0.16	*ACT*	2.08 ± 0.43	*PP2C*	0.99	*FAB1A*	1.86
	2	*PPR*	0.45	*FAB1A*	0.26	*PPR*	2.32 ± 0.6	*FAB1A*	1.03	*PP2C*	2.00
	3	*HLMt*	0.46	*HLMt*	0.37	*FAB1A*	3.12 ± 0.81	*HLMt*	1.08	*PPR*	2.51
	4	*PP2C*	0.48	*Fbp2*	0.44	*PP2C*	3.46 ± 0.86	*PPR*	1.14	*HLMt*	3.41
	5	*Fbp3*	0.52	*PPR*	0.48	*HLMt*	3.54 ± 0.93	*Fbp2*	1.16	*ACT*	4.45
	6	*Fbp2*	0.59	*Fbp3*	0.49	*Fbp3*	3.81 ± 0.99	*Fbp3*	1.18	*Fbp2*	5.38
	7	*ACT*	0.65	*ODD*	0.62	*Fbp2*	4.04 ± 1.07	*ACT*	1.33	*Fbp3*	5.73
	8	*ODD*	0.80	*ACT*	0.68	*CSA*	4.56 ± 1.12	*ODD*	1.33	*ODD*	7.97
	9	*S8*	0.92	*CCS1*	0.70	*ODD*	5.28 ± 1.4	*CCS1*	1.44	*CCS1*	9.49
	10	*CCS1*	1.01	*S8*	0.85	*CCS1*	5.35 ± 1.52	*S8*	1.55	*S8*	9.97
	11	*UK*	1.15	*UK*	1.1	*S8*	5.77 ± 1.66	*UK*	1.83	*CSA*	10.84
	12	*CSA*	1.27	*CSA*	1.19	*Fbp1*	7.69 ± 2.23	*CSA*	1.93	*UK*	11.47
	13	*Fbp1*	1.38	*Fbp1*	1.20	*UK*	8.16 ± 2.26	*Fbp1*	1.97	*Fbp1*	12.74
**SA**	1	*FAB1A*	0.17	*PPR*	0.14	*ODD*	0.97 ± 0.24	*ODD*	0.48	*ODD*	2.00
	2	*Fbp3*	0.17	*Fbp1*	0.15	*Fbp1*	1.18 ± 0.3	*Fbp1*	0.49	*Fbp1*	2.63
	3	*CCS1*	0.18	*PP2C*	0.16	*CCS1*	1.24 ± 0.34	*PP2C*	0.50	*Fbp3*	3.20
	4	*ODD*	0.24	*ODD*	0.18	*Fbp3*	1.36 ± 0.32	*PPR*	0.51	*PPR*	3.87
	5	*UK*	0.29	*Fbp2*	0.22	*UK*	1.48 ± 0.44	*Fbp3*	0.51	*FAB1A*	4.56
	6	*Fbp1*	0.32	*HLMt*	0.32	*FAB1A*	1.66 ± 0.4	*Fbp2*	0.54	*PP2C*	5.01
	7	*PP2C*	0.34	*Fbp3*	0.35	*PPR*	1.68 ± 0.41	*UK*	0.55	*CCS1*	5.89
	8	*PPR*	0.36	*UK*	0.35	*HLMt*	1.87 ± 0.46	*FAB1A*	0.56	*UK*	6.88
	9	*Fbp2*	0.39	*FAB1A*	0.42	*ACT*	1.98 ± 0.39	*HLMt*	0.59	*Fbp2*	7.38
	10	*HLMt*	0.41	*CCS1*	0.48	*Fbp2*	2.26 ± 0.55	*CCS1*	0.59	*HLMt*	8.35
	11	*ACT*	0.44	*CSA*	0.50	*PP2C*	2.37 ± 0.54	*CSA*	0.71	*ACT*	9.43
	12	*CSA*	0.48	*ACT*	0.67	*CSA*	2.59 ± 0.62	*ACT*	0.73	*CSA*	11.49
	13	*S8*	0.63	*S8*	1.47	*S8*	4.61 ± 1.37	*S8*	1.48	*S8*	13.00
**MeJA**	1	*PP2C*	0.08	*CSA*	0.05	*Fbp3*	0.26 ± 0.06	*CSA*	0.18	*CSA*	1.19
	2	*CSA*	0.08	*Fbp3*	0.08	*CSA*	0.27 ± 0.06	*Fbp3*	0.19	*Fbp3*	1.86
	3	*Fbp3*	0.09	*ODD*	0.10	*Fbp2*	0.28 ± 0.07	*PP2C*	0.20	*PP2C*	2.63
	4	*Fbp2*	0.10	*PP2C*	0.11	*PPR*	0.37 ± 0.09	*ODD*	0.21	*Fbp2*	4.16
	5	*ODD*	0.12	*Fbp2*	0.12	*PP2C*	0.4 ± 0.09	*Fbp2*	0.21	*ODD*	4.36
	6	*PPR*	0.12	*PPR*	0.13	*ODD*	0.42 ± 0.1	*PPR*	0.22	*PPR*	5.73
	7	*Fbp1*	0.13	*Fbp1*	0.16	*S8*	0.44 ± 0.12	*Fbp1*	0.23	*Fbp1*	7.24
	8	*ACT*	0.15	*ACT*	0.17	*Fbp1*	0.49 ± 0.12	*ACT*	0.24	*ACT*	7.74
	9	*S8*	0.16	*S8*	0.17	*ACT*	0.54 ± 0.11	*S8*	0.25	*S8*	9.00
	10	*CCS1*	0.18	*CCS1*	0.23	*UK*	0.56 ± 0.16	*CCS1*	0.28	*CCS1*	10.24
	11	*UK*	0.20	*UK*	0.25	*CCS1*	0.61 ± 0.17	*UK*	0.31	*UK*	10.74
	12	*HLMt*	0.23	*HLMt*	0.32	*HLMt*	1.3 ± 0.32	*HLMt*	0.36	*HLMt*	12.00
	13	*FAB1A*	0.25	*FAB1A*	0.35	*FAB1A*	1.4 ± 0.34	*FAB1A*	0.39	*FAB1A*	13.00
**GA**	1	*PP2C*	0.12	*Fbp2*	0.09	*CCS1*	0.6 ± 0.16	*Fbp2*	0.23	*PP2C*	1.57
	2	*CCS1*	0.12	*UK*	0.13	*PP2C*	0.64 ± 0.14	*PP2C*	0.24	*Fbp2*	2.59
	3	*HLMt*	0.16	*PP2C*	0.14	*S8*	0.91 ± 0.24	*UK*	0.25	*CCS1*	2.66
	4	*UK*	0.19	*HLMt*	0.16	*UK*	0.94 ± 0.28	*HLMt*	0.25	*UK*	3.60
	5	*Fbp2*	0.19	*CCS1*	0.17	*Fbp1*	1.11 ± 0.27	*CCS1*	0.26	*HLMt*	3.94
	6	*CSA*	0.21	*CSA*	0.19	*HLMt*	1.11 ± 0.27	*CSA*	0.27	*CSA*	6.82
	7	*PPR*	0.22	*PPR*	0.19	*FAB1A*	1.19 ± 0.29	*Fbp3*	0.27	*S8*	7.40
	8	*Fbp3*	0.22	*Fbp3*	0.19	*Fbp2*	1.22 ± 0.29	*PPR*	0.27	*PPR*	8.10
	9	*Fbp1*	0.23	*Fbp1*	0.21	*ACT*	1.39 ± 0.26	*Fbp1*	0.29	*Fbp1*	8.13
	10	*S8*	0.24	*S8*	0.22	*CSA*	1.45 ± 0.33	*S8*	0.29	*Fbp3*	8.56
	11	*FAB1A*	0.25	*FAB1A*	0.25	*PPR*	1.54 ± 0.36	*FAB1A*	0.29	*ACT*	9.12
	12	*ACT*	0.26	*ACT*	0.28	*Fbp3*	1.64 ± 0.38	*ACT*	0.33	*FAB1A*	10.16
	13	*ODD*	0.28	*ODD*	0.33	*ODD*	1.9 ± 0.46	*ODD*	0.37	*ODD*	13.00
**Total hormone**	1	*PPR*	0.22	*Fbp2*	0.12	*UK*	0.96 ± 0.28	*Fbp1*	0.47	*Fbp1*	1.73
	2	*Fbp2*	0.22	*PPR*	0.14	*Fbp1*	1.09 ± 0.27	*Fbp2*	0.48	*Fbp2*	1.78
	3	*Fbp1*	0.26	*Fbp1*	0.16	*Fbp3*	1.36 ± 0.32	*PPR*	0.49	*PPR*	2.55
	4	*HLMt*	0.30	*HLMt*	0.24	*ODD*	1.41 ± 0.35	*HLMt*	0.53	*UK*	4.53
	5	*UK*	0.35	*PP2C*	0.34	*FAB1A*	1.46 ± 0.35	*ODD*	0.54	*HLMt*	5.03
	6	*ODD*	0.37	*ODD*	0.35	*Fbp2*	1.46 ± 0.35	*UK*	0.55	*ODD*	5.18
	7	*Fbp3*	0.39	*UK*	0.37	*CCS1*	1.49 ± 0.41	*CCS1*	0.55	*Fbp3*	6.24
	8	*CCS1*	0.40	*CCS1*	0.37	*PPR*	1.62 ± 0.39	*Fbp3*	0.56	*CCS1*	7.74
	9	*FAB1A*	0.41	*Fbp3*	0.44	*HLMt*	1.86 ± 0.45	*PP2C*	0.58	*PP2C*	8.59
	10	*ACT*	0.42	*ACT*	0.46	*ACT*	2.15 ± 0.41	*ACT*	0.60	*FAB1A*	9.19
	11	*PP2C*	0.44	*CSA*	0.47	*PP2C*	2.23 ± 0.5	*FAB1A*	0.61	*ACT*	9.74
	12	*CSA*	0.48	*FAB1A*	0.49	*CSA*	2.77 ± 0.64	*CSA*	0.67	*CSA*	11.74
	13	*S8*	0.61	*S8*	1.34	*S8*	4.63 ± 1.3	*S8*	1.36	*S8*	13.00
**Total**	1	*HLMt*	0.37	*HLMt*	0.31	*CCS1*	3.13 ± 0.86	*HLMt*	0.91	*HLMt*	1.50
	2	*PPR*	0.37	*PPR*	0.35	*FAB1A*	3.16 ± 0.78	*PPR*	0.92	*PPR*	1.86
	3	*FAB1A*	0.48	*FAB1A*	0.41	*PPR*	3.52 ± 0.86	*FAB1A*	0.95	*FABIA*	2.71
	4	*Fbp2*	0.53	*Fbp2*	0.48	*HLMt*	3.59 ± 0.9	*Fbp2*	0.97	*ACT*	4.45
	5	*Fbp3*	0.55	*PP2C*	0.54	*ACT*	3.59 ± 0.71	*PP2C*	1.00	*Fbp2*	4.76
	6	*PP2C*	0.57	*ODD*	0.54	*ODD*	3.87 ± 0.97	*Fbp3*	1.03	*PP2C*	6.37
	7	*ACT*	0.61	*ACT*	0.63	*UK*	3.91 ± 1.13	*ODD*	1.04	*Fbp3*	6.40
	8	*ODD*	0.66	*Fbp3*	0.64	*Fbp3*	4.43 ± 1.08	*ACT*	1.05	*ODD*	6.70
	9	*CCS1*	0.73	*CCS1*	0.68	*Fbp2*	4.44 ± 1.1	*CCS1*	1.14	*CCS1*	7.35
	10	*CSA*	0.81	*CSA*	1.00	*CSA*	4.65 ± 1.1	*CSA*	1.32	*CSA*	9.74
	11	*Fbp1*	0.94	*S8*	1.38	*S8*	5.44 ± 1.54	*S8*	1.63	*S8*	11.49
	12	*S8*	1.05	*Fbp1*	1.52	*PP2C*	5.51 ± 1.27	*Fbp1*	1.70	*Fbp1*	11.98
	13	*UK*	1.21	*UK*	1.98	*Fbp1*	6.64 ± 1.74	*UK*	2.11	*UK*	12.17

**Figure 2:**
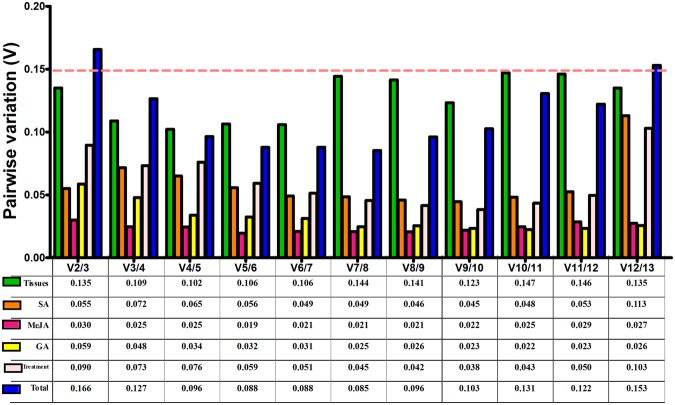
Pairwise variation (V) analysis of 13 selected reference genes using geNorm software. The pairwise variations Vn/Vn + 1 were calculated by geNorm in different tissues and under hormone treatment samples.

The geNorm program was also performed to determine the optimal number of reference genes for normalizing RT-qPCR data by calculating the pairwise variations Vn/Vn + 1. As shown in Fig. 2, the value of V2/3 was always below the cut-off value of 0.15 in different tissue samples and samples from all of the hormone treatments, indicating that the two most stable reference genes were sufficient to normalize expression data in these experiments. In the total experimental samples, the three most stable reference genes were ideal to normalize RT-qPCR data since the value of V3/4 (0.127) was below the cut off value of 0.15.

The results calculated with NormFinder (Table 2) show that *PP2C* followed by *FAB1A* were the most stable genes in all tested tissues, *Fbp1* was also considered to be a weakly stable gene in such a sample set. As for SA treatment, *PPR* and *Fbp1* were the most stable reference genes. *CSA* and *Fbp3* were the top ranked reference genes in MeJA treatment samples. *Fbp2* and *UK* were the most stable reference genes for GA treatment. *Fbp2* and *PPR* were the most highly ranked reference genes in all three hormone treatment samples. When assessing the total experimental samples, *HLMt* and *PPR* were the top stably expressed genes.

As shown in Table 2, when evaluated by the BestKeeper program, *ACT* followed by *PPR* were the most stable genes and *UK* was considered to be the least stable gene in the four tissue samples tested. *ODD* and *Fbp1* for the SA treatment, *Fbp3* and *CSA* for the MeJA treatment, *CCS1* and *PP2C* for the GA treatment, as well as *UK* and *Fbp1* for all three hormone treatments were the most stable reference genes. As for the total experimental samples, *CCS1* and *FAB1A* were the top stably expressed reference genes.

According to the ranking orders generated by Delta Ct (Table 2), *PP2C* and *FAB1A* were the most stable genes and *Fbp1* was the least stable gene in the total of four tissues tested. As for hormone treatment, *ODD* and *Fbp1* for SA treatment, *CSA* and *Fbp3* for MeJA treatment, *Fbp2* and *PP2C* for GA treatment, and *Fbp1* followed by *Fbp2* for all three hormone treatments were the top ranking reference genes. *HLMt* and *PPR* were the most stable reference genes for the total of experimental samples.

Finally, RefFinder was used to comprehensively validate the stability of candidate reference genes. According to the results determined by RefFinder (Table 2) and geNorm (Fig. 2), the combination of *FAB1A* and *PP2C* for all four tissues tested, *ODD* and *Fbp1* for SA treatment, *CSA* and *Fbp3* for MeJA treatment, *PP2C* and *Fbp2* for GA treatment, as well as *Fbp1* and *Fbp2* for the total of three hormone treatments were the most suitable reference genes. As for all of the experimental samples, the most suitable reference genes were the combination of *HLMt*, *PPR* and *FAB1A*.

**Figure 3:**
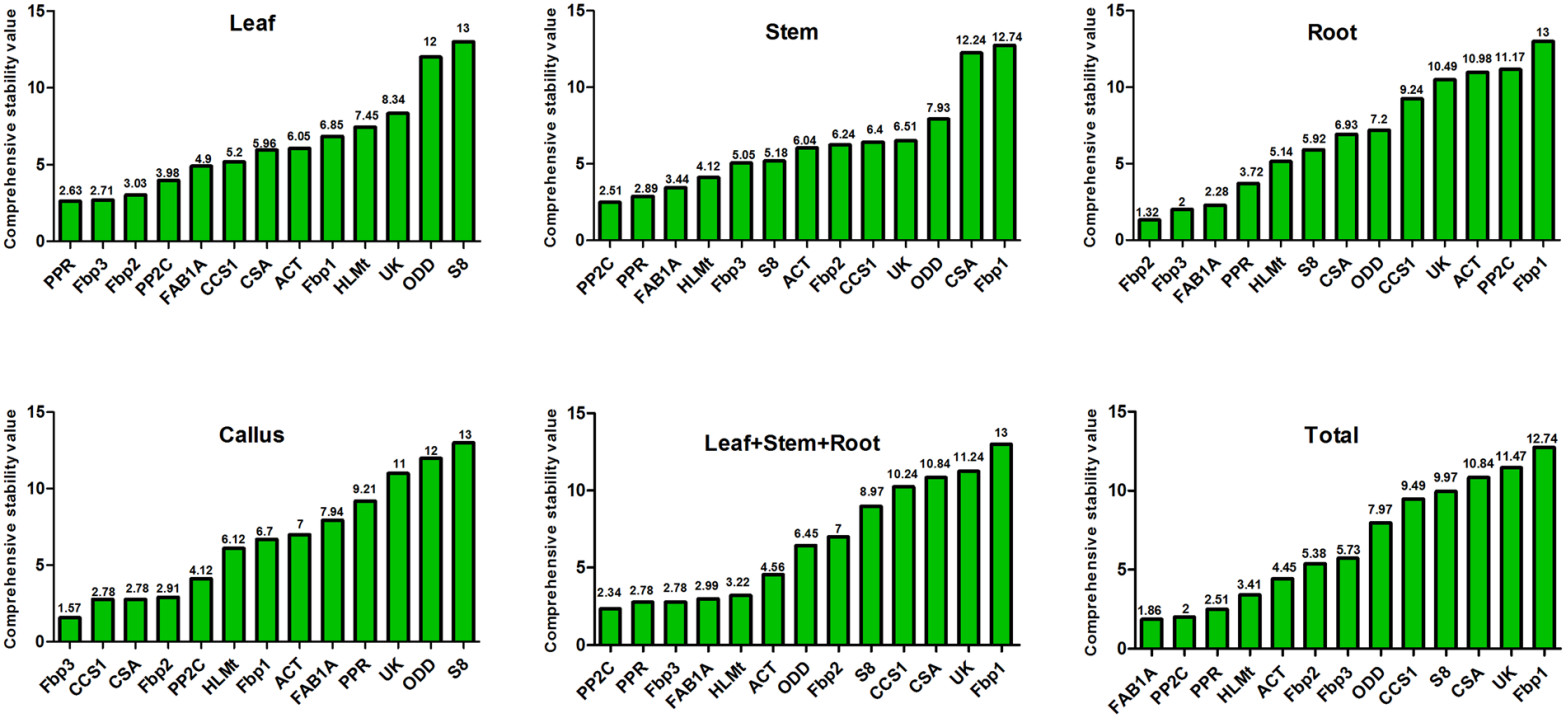
Comprehensive expression stability of 13 selected reference genes recommended by RefFinder in specific tissues and different tissue combinations.

Moreover, we also verified the stability of candidate reference genes in specific tissues and different tissue combinations using RefFinder. According to the comprehensive ranking recommended by RefFinder (Fig. 3), *PPR* and *Fbp3* were the most stable reference genes in leaves, *Fbp2* and *Fbp3* were the most stable genes in roots, and *Fbp3*, *CCS1* and *CSA* were the most stable genes in callus. In stems as well as the combination of leaf, stem and root (LSR), *PP2C* and *PPR* were the most stable reference genes. *Fbp3* was also among the most stable reference genes in LSR.

### Validation of Identified reference genes in different Tissues and under MeJA treatment

In order to validate the identified reference genes, the transcript profile of a key gene (*SaSSy*) was investigated in the reference genes, and evaluated in the three tissues and under MeJA treatment.

**Figure 4:**
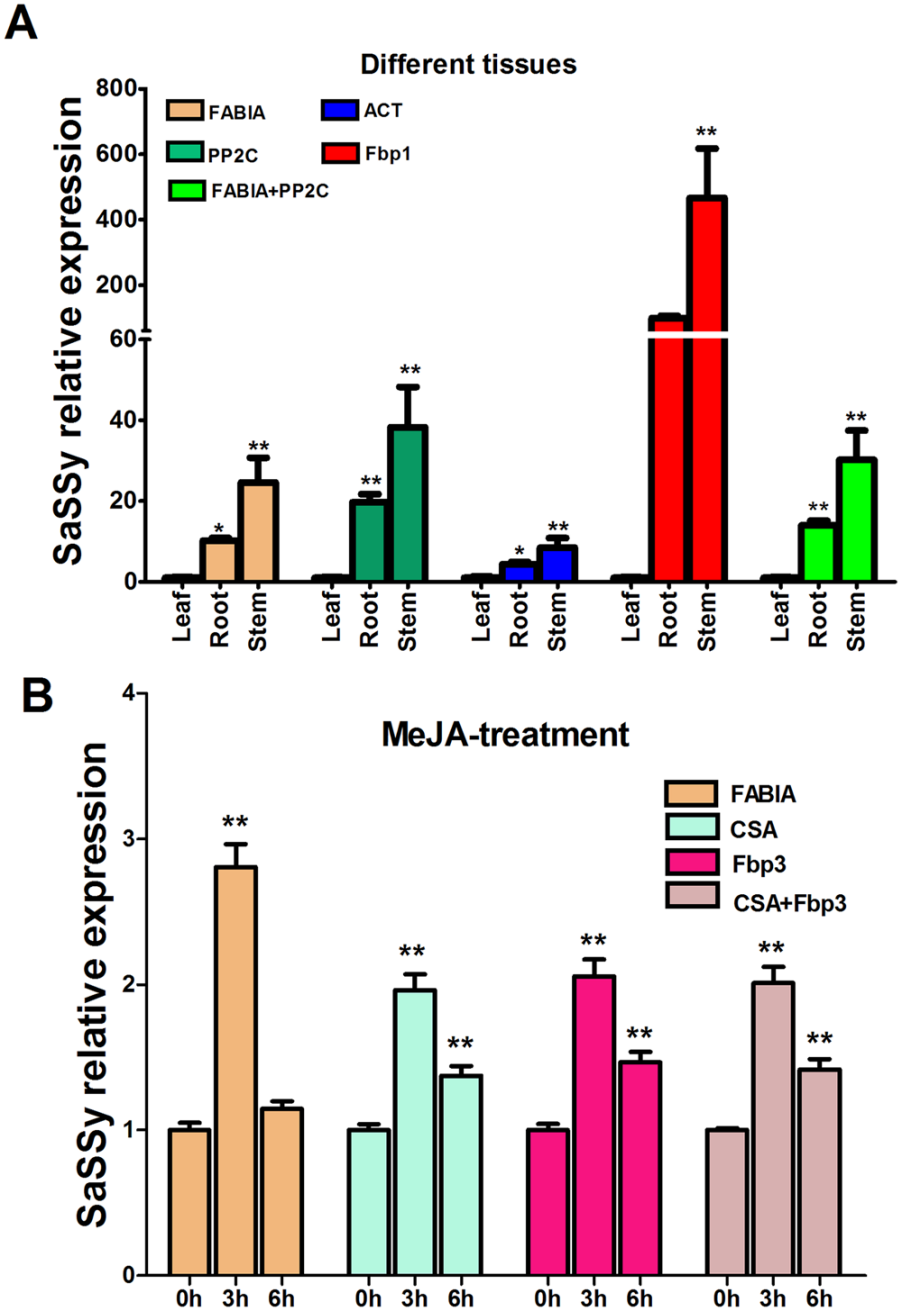
Relative expression levels of the *SaSSy* gene normalized by a validated reference gene alone or combination in different tissues (**A**) and under MeJA treatment (**B**) of *Santalum album*. Bars indicate standard deviation calculated from three biological replicates. Asterisk indicates significance at *P* < 0.05(*) or *P* < 0.01(**) using Duncan’s multiple range test.

As shown in Fig. 4A, the expression level of the *SaSSy* gene was similar in the three tested tissues when using the two most stable reference genes (*FAB1A* and *PP2C*) to normalize RT-qPCR data. The combination of *FAB1A* + *PP2C* provides more accurate expression values for each tissue than a single reference gene. Although each of the reference genes (*FAB1A*, *PP2C*, *ACT* and *Fbp1*) and gene combination (*FAB1A* + *PP2C*) used for normalization provided a similar trend of *SaSSy* expression level (leaf < root < stem) (this trend was comparable with the RNA-seq result), when the least stable reference gene *Fbp1* was used to normalize RT-qPCR data, the expression level of *SaSSy* was obviously over-estimated in tested tissues. Statistical analysis showed insignificantly different results in roots when normalized by *Fbp1*, so it generated inconsistent statistical results compared with the results normalized by more stable reference genes or their combination. These over-estimated results, especially in roots, did not match RNA-seq results. Furthermore, *SaSSy* expression level was considerably reduced in all tissues when the traditional housekeeping gene (*ACT*) was used for normalization.

Under the MeJA treatment, the expression of *SaSSy* at 3 h is about 2.0 times higher than no treatment control (0 h) when normalized by the most stable reference genes (*CSA*, *Fbp3*) and their combination (*CSA* + *Fbp3*). While using the least stable gene *FAB1A*, it was more than 2.8 times higher. The *SaSSy* expression at 6 h is about 1.4 times higher than at 0 h using the best reference genes (*CSA*, *Fbp3*, *CSA* + *Fbp3*). However, the least stable reference gene *FAB1A* produced an obviously reduced and statistically insignificant reduction of 1.15 times (Fig. 4B). This demonstrated an obviously effect of using different reference genes for normalization.

## Discussion

A powerful technique, RT-qPCR, has been widely used for the detection and quantification of gene expression in plants. In order to interpret RT-qPCR data accurately and reliably, appropriate reference genes are essential. Many reports in several plant species such as *Arabidopsis* (*Arabidopsis thaliana*), tomato (*Solanum lycopersicum*) and grape (*Vitis vinifera*) have shown the importance and need to validate appropriate reference genes for normalizing RT-qPCR data in different tissues, developmental stages or experimental conditions^22–27^. To date, only the housekeeping gene *ACT* was used as a reference gene in *S*. *album*, and no systematic validation and evaluation of stable reference genes for RT-qPCR data normalization exists for the commercial *S*. *album* tree. In this study, we took advantage of previously published data^12,13,15^ to select stable reference genes in different tissues of *S*. *album*. A total of 12 novel genes were selected as candidate reference genes based on the CV value calculated from transcriptome data. These genes had an intermediate or low level of expression according to the mean expression values (MVs) (Supplementary Table S1), similar to Czechowski *et al*.^22^. In order to compare the stability of these 12 candidate reference genes, the currently used housekeeping gene *ACT* for *S*. *album* was also included in our analysis.

In general, multiple methods should be employed to validate the stability of reference genes to avoid artificial results. The most widespread methods used are geNorm, NormFinder and BestKeeper. In this study, the ranking of stability of the 13 candidate reference genes, as arranged by geNorm and NormFinder (Table 2), was rather similar. As can be seen in different tissues tested (Table 2), *FAB1A* was ranked in first position by geNorm and second by NormFinder, while *Fbp1* was always ranked 13th based on both methods. However, BestKeeper generated a different ranking. For instance, *ACT* was ranked first by BestKeeper but in a medium position by geNorm and NormFinder in different tissues. Similar results could be found in other sample sets. Previous studies also demonstrated similar results in *Taihangia rupestris* flowers^28^, under hormone stress in *Brassica napus*^29^, and under heat stress as well as in all samples including various abiotic stresses, tissues and ages of *Salicornia europaea*^30^. This is because different algorithm models and statistical methods are used in each software. In order to obtain a global and comprehensive result, the integrative RefFinder web-based tool is widely used to arrange the final ranking of reference genes, such as in tissue development in kenaf (*Hibiscus cannabinus*)^31^, under abiotic stresses in creeping bentgrass (*Agrostis stolonifera*)^32^ as well as in Seashore paspalum (*Paspalum vaginatum*)^33^. Based on RefFinder analysis (Table 2), some novel genes were identified as the most stably expressed reference genes, i.e., *FAB1A* for all tissues tested, *ODD* for SA treatment, *CSA* for MeJA treatment, and *HLMt* for all experimental samples were the best stable reference genes. *PP2C*, a conserved serine/threonine protein phosphatase gene, was the most stable reference gene for GA treatment, and previous studies showed that another conserved serine/threonine protein phosphatase 2 A gene (*PP2A*) was the most stable reference gene in root tissue and hormone treatments of garden pea (*Pisum sativum*)^34^, both in diurnal and developmental time-course experiments in lettuce (*Lactuca sativa*)^35^, in total samples (including five different tissues and three different abiotic stresses) and under abiotic stress treatments in *Isatis indigotica*^36^, as well as under various stresses in rapeseed (*Brassica napus*)^37^. One of the best reference genes for all experimental samples, *PPR*, is one of the largest gene superfamilies and is essential in mitochondria and chloroplasts biogenesis, plastid gene expression, mitochondrial RNA editing, as well as early embryogenesis in plants^38–41^. Previous studies selected and validated several *PPR* superfamily genes as the most stable reference genes in *B*. *napus*^29^ and *Arabidopsis thaliana*^22^. The F-box family gene *Fbp1*, *Fbp2* and *Fbp3* were also among the most stable reference genes for SA and total hormone treatment, for GA treatment, and for MeJA treatment samples, respectively. In contrast, *Fbp1* was also identified as the least stable gene among all four *S*. *album* tissues tested. A previous study demonstrated that the F-box gene was the most stable expression reference gene in *B*. *napus*^37^ and soybean (*Glycine max*)^42^, but was the least stably expressed gene in different tissues of licorice (*Glycyrrhiza glabra*) under drought stress^43^. This could be explained by the fact that the F-box protein is one of the largest and most heterogeneous superfamilies in plants, and plays a wide range of roles in plant growth and development^44^. Similar results were also found in our newly selected reference genes *FAB1A* and *ODD* (Table 2). Therefore, the expression of a reference gene within the same gene family can vary in different tissues and experimental conditions. For this reason, it is essential to select and validate reference genes for specific tissue samples and experimental conditions.

There is no universally stable reference gene for all experiments, and multiple reference genes should be used to obtain more accurate and reliable results^2,3,16^. In this study, we examined the interference of the most and least stable reference gene and their combination on the expression level of the *SaSSy* gene. In different tissues (Fig. 4A), the expression data of *SaSSy* normalized by the most stable genes *FAB1A* and *PP2C* was similar, but the data normalized by the combination of *FAB1A* and *PP2C* was more precise. When the least stable reference gene *Fbp1* was used as the normalizer, the expression level of *SaSSy* was fully overestimated in tested tissues. Under MeJA treatment (Fig. 4B), the expression level of *SaSSy* presented a significant difference when the most and least stable reference genes were used for normalization. Results were identical when the two best reference genes alone or their combination were used for normalization. However, the least stable gene *FAB1A* not only overestimated *SaSSy* expression level at 6 h but also significantly reduced the expression at 3 h, and thus generated an incorrect interpretation of the results. Thus, for all four tissues, we recommend that the two most stable genes (*FAB1A* and *PP2C*) are ideal for normalization. As for hormone treatment, we suggest that one most stable reference gene could be sufficient to normalize RT-qPCR data.

There is increasing evidence that more stable reference genes can be selected using high-throughput transcriptomic data^22,26,42,45^. Our current study also demonstrates that several novel reference genes (*FAB1A*, *CSA*, *ODD*, *PP2C*, *PPR* and *HLMt*), which were selected from a set of RNA-seq data of *S*. *album*, performed better than the traditional housekeeping gene *ACT*. Indeed, when the RT-qPCR data was normalized by *ACT*, the expression level of *SaSSy* decreased and exhibited inconspicuous and inaccurate discrepancies among all tested tissue samples of *S*. *album* (Fig. 4A). Zhou *et al*.^46^ also demonstrated that unstably higher expression level of reference genes lowered the expression of the target gene and caused indistinguishable discrepancies in different species of oil-tea (*Camellia sinensis*). Therefore, our study confirmed the feasibility of reference gene selection using high-throughput transcriptome data. However, due to variation in the expression level of different samples and experimental conditions, reference genes selected based on RNA-seq data should be further validated when the samples and conditions are different from RNA-seq data. Furthermore, we recommend that transcriptome data be employed from diverse samples and conditions to obtain more universally stable reference genes.

In conclusion, for the first time in sandalwood research, this study has systematically selected and evaluated appropriate reference genes for RT-qPCR in four tissues (stem, root, leaf and callus) and the tissue of callus under three hormone treatments based on RNA-seq data. A total of 13 candidate reference genes were selected then verified using geNorm, NormFinder, BestKeeper, Delta Ct and RefFinder software, and the results was further validated by *SaSSy* gene expression analysis in different tissues and under MeJA treatment. The combination of *FAB1A* and *PP2C* was the optimal combination of reference genes for all tissues tested, while *Fbp1* and *Fbp2* were the most suitable reference genes under three hormone treatments. Our results demonstrated that the suitable reference genes for RT-qPCR normalization are not identical in different experimental samples, so optimal reference genes or their combinations should be selected according to specific experimental conditions. The stable reference genes obtained in this study will undoubtedly improve the accuracy of RT-qPCR data normalization and quantification under hormone treatment as well as in different tissues of *S*. *album* and facilitate functional gene analysis in sandalwood in the future.

## Materials and Methods

### Collection of plant materials and hormone treatments

The leaves, roots and stems were collected from three 5- to 7-year-old *S*. *album* tress growing in the South China Botanical Garden in April, 2017. The stem was prepared as shavings using a hand-driven drill at 20, 40 and 60 cm from the ground, and then pooled as the stem sample for each tree.

Callus was induced as follows: newly sprouting shoots from trees were selected as the explant, their surface was swabbed clean with cotton dipped in 75% alcohol, then immersed into 0.1% (w/v) mercuric chloride for 15–20 min, followed by five successive washes in sterile distilled water. Shoot segments with a single node were inoculated vertically on solid Murashige and Skoog (MS) basal medium^47^ to which 1.0 mg/L thidiazuron (TDZ) was added to induce callus. Cultures were incubated at 25 ± 1 °C under a 16-h photoperiod provided by cool white fluorescent lamps with a light intensity of approximately 50 µmol m^−2^ s^−1^. The resulting callus was proliferated on solid MS medium supplemented with 1.5 mg/L 2,4-dichlorophenoxyacetic acid (2,4-D) and 0.2 mg/L TDZ. After about 20 days, similarly good callus was collected as callus tissue samples and used for hormone treatment. Callus was frozen immediately in liquid nitrogen (N_2_) and stored at −80 °C until use. Each tissue (stem, leaf, root, callus clump) sample collected or generated from a tree was used as a biological replicate. Three biological replicates were used for each sample.

Similarly good callus proliferated after transfer into liquid Murashige and Skoog (MS) basal medium^47^ supplemented with 1.5 mg/L 2,4-D and 0.2 mg/L TDZ, and then placed on a shaker at 100 rpm/min in the same culture conditions mentioned above. After 24 h, dissolved SA, MeJA and GA were added separately into samples at a final concentration of 100 µM for each hormone treatment, and all samples were collected at 0, 3 and 6 h as triplicates. All samples were frozen immediately in N_2_ and stored at −80 °C until use.

### RNA extraction and cDNA synthesis

RNA extraction and RT-qPCR experiments were carried out on the basis of the Minimum Information for Publication of Quantitative Real-Time PCR Experiments (MIQE) guideline^48^ to ensure the reliability of results. Total RNA was isolated from all samples using a protocol reported for the isolation of RNA from woody plants^49^. To remove DNA, the total RNA of all samples was digested with RNase-free DNase I (Takara, Dalian, China) at 37 °C for 30 min according to the manufacturer’s instructions. Then, PCR of the *ACT* gene was conducted using RNA as the template in 40 cycles to determine DNA contamination with specific primers (F: AGGCTGTTCTTTCCCTTTA, R: TTCCTTGCTCATTCTATCG). The DNA-free total RNA was qualified and quantified using a NanoDrop ND-1000 spectrophotometer (Nanodrop Technologies, Wilmington, NC, USA). The RNA samples with an A_260_/A_280_ ratio between 1.9 and 2.1, and an A_260_/A_230_ ratio greater than 2.0 were used for subsequent analysis. RNA integrity was assessed by 1.0% (w/v) agarose gel electrophoresis, and confirmed with an Agilent 2100 Bioanalyzer. Total RNA (1 µg) was used to synthesize first strand cDNAs using an equivalent of oligo-(dT)_15_ and random primers with a GoScript™ Reverse Transcriptase system (Promega, Madison, WI, USA) in 20 µL volume according to the manufacturer’s protocols. Amplification was performed at 25 °C for 5 min, 42 °C for 60 min, and 70 °C for 15 min. The successfully synthesized cDNA samples were diluted 1:10 with nuclease-free water and stored at −20 °C until further use.

### Selection of candidate reference genes using RNA-seq data and design of primers

The transcriptome sequencing data from wood in stems^13^ (GenBank Accession: PRJNA297453), roots^12^ (GenBank Accession: SRA150639) and leaves^15^ (GenBank Accession: SRR3731808, SRR3731809) of *S*. *album* were used to select the most stably expressed genes. To estimate the expression stability of every gene, we analyzed all the raw data for each gene using the method described by Wang *et al*.^50^, noted briefly as follows: (a) reads per kb per million reads (RPKM) values, mean expression values (MVs), and standard deviations (SDs) for each gene were calculated; (b) the coefficient of variation (CV) of each gene was calculated using the formula CV = SD/MV × 100%; (c) all genes were ranked based on their CV value. In general, gene expression is more stable while its CV value is much lower. Based on this principal and the method described by de Jonge *et al*.^51^, candidate reference genes that met the following requirements were selected: (a) maximum fold change (MFC) < 2; (b) mean value of FPKM of any gene pairs < (maximum expression value − 2 × SD) in the dataset; (c) a CV ≤ 12%.

RT-qPCR primers were designed using primer3 plus (http://www.bioinformatics.nl/cgi-bin/primer3plus/primer3plus.cgi). Primer design considered the following criteria: (a) primer size: 20–23 bp; (b) product size: 80–200 bp; (c) GC% content: 40–60% (primers are shown in Table 1). Moreover, primer accuracy and specificity were checked by 2.0% (w/v) agarose gel electrophoresis. The melting curve and no template control (NTC) were prepared to further validate the specificity and absence of primer dimer formation and DNA contamination for every primer pair.

### Quantitative real-time PCR (qPCR) and amplification efficiency

RT-qPCR was performed in 96-well plates in an ABI 7500 Real-time system (ABI, Alameda, CA, USA) using the SYBR Premix Ex Taq™ Kit (Takara). The qPCR reaction in a total volume of 10 µL consisted of 5 µL SYBR Premix Ex Taq (1×), 500 nM of each forward and reverse primer, 100 ng of cDNA, and 3 µL of ddH_2_O. The cycling conditions were: 95 °C for 2 min, followed by 40 cycles at 95 °C for 15 s and 60 °C for 1 min. After 40 cycles, a melting curve analysis was performed ranging from 60 to 95 °C.

A standard curve was established by triplicate repeats of RT-qPCR amplification using serial dilutions (1:1, 1:10, 1:100, 1:1000, and 1:10000) of all tested cDNA sample pools. The correlation coefficient (*R*^2^) and amplification efficiency (*E*) for each gene were calculated based on the standard curve. The amplification efficiency of each gene was calculated using the equation *E* = (10^–1/slope^ – 1) × 100%^48^. All RT-qPCR experiments were carried out using three biological replicates of each sample, as indicated above, and three technical replicates of each biological replicate.

### Gene stability analysis

Five different programs, geNorm (version 3.5)^16^, NormFinder^17^, BestKeeper^18^, Delta Ct^19^ and RefFinder^20^, a web-based tool (http://150.216.56.64/referencegene.php), were used to determine the stability of candidate reference genes. For the geNorm and NormFinder algorithms, the raw Cq data from each sample was converted into relative quantity (RQ) using the formula 2^ΔCq^, where ΔCq = min Cq (of each gene) – sample Cq. The GeNorm program first calculates an expression stability (*M*) value for each gene and then calculates a pairwise variation (Vn/n + 1) value between genes. Genes with an *M* value below 1.5 are supposed to be stably expressed, and a lower *M* value indicates a more stable level of expression^16^. Moreover, optimal number of reference genes for normalization is indicated. The value of “n” is the optimal number of reference genes when the pairwise value of variation (Vn/Vn + 1) is below a cut-off value of 0.15^16^. NormFinder was used to rank the stability of candidate reference genes with an *M* value that took into account the value of inter- and intra-group variance. Genes with the lowest stability value indicate the most stable expression within the examined gene set^17^. The BestKeeper program examines the ranking of reference genes based on the calculation of the CV and SD values for each gene. Any gene with an SD value less than 1.0 was recommended as a gene with stable expression while genes with both the lowest CV and SD values represent the highest stability in the BestKeeper program^18^. The Delta Ct approach compares the difference in Cq values of reference genes pairwise and ranks the candidate reference genes using the variability of averaged SD^19^. RefFinder integrates the three classical algorithms (geNorm, NormFind and BestKeeper) and a comparative ΔCt method to comprehensively validate and rank the stability of candidate reference genes by calculating the geometric mean of their weights for the overall final ranking^20^.

### Validation of reference genes

To validate the reliability of the selected reference genes (including the least and most stable reference genes and their combinations) recommended by the RefFinder tool, the expression level of *SaSSy*, a key gene for synthesizing α-, β-, and *epi*-β-santalene (the precursor of the main component of *S*. *album* oil (*Z*)-α-santalol and (*Z*)-β-santalol)^9–11^, was determined in stem, leaf, and root tissues and the tissue of callus under MeJA treatment at different times using the 2^-ΔΔCq^ method^52^. The primer for *SaSSy* was designed according to the criteria mentioned above. Specificity was checked as described above (primer pairs are shown in Table 1).

### Statistical analysis

The Kolmogorov-Smirnov test, which is used to verify data normality with a KS-test p value, was performed in SPSS13.0 (SPSS Inc., Chicago, IL, USA). Data with a KS-test p value > 0.05 was considered as normal. Following one-way analysis of variance (ANOVA), significant differences were assessed by Duncan’s multiple range test at *P* < 0.05 (*) and *P* < 0.01 (**).

## Electronic supplementary material

Dataset 1

## Data Availability

All data generated or analyzed during this study are included in this published article (and its Supplementary Information files).
