# Supplementary material for: Selection and Validation of Novel RT-qPCR Reference Genes under Hormonal Stimuli and in Different Tissues of Santalum album
Source: Sci Rep. 2018 Nov 30;8:17511. doi: 10.1038/s41598-018-35883-6 (PMC6269485; doi:10.1038/s41598-018-35883-6)
Supplement: Supplementary file 1 — Dataset 1 [file 41598_2018_35883_MOESM1_ESM.pdf]

Selection and Validation of Novel RT-qPCR Reference Genes under Hormonal Stimuli and in Different Tissues of *Santalum album*

Haifeng Yan[1, 2], Yueya Zhang[1, 2], Yuping Xiong[1, 2], Qingwei Chen[1], Hanzhi Liang[1], Meiyun Niu[1,2], Beiyi Guo[1,2], Mingzhi Li[4], Xinhua Zhang[1*], Yuan Li[1], Jaime A. Teixeira da Silva[3*], Guohua Ma[1*]

1 Guangdong Provincial Key Laboratory of Applied Botany, South China Botanical Garden, the Chinese Academy of Sciences, Guangzhou, 510650, China

2 University of Chinese Academy of Sciences, Beijing 100039, China

3 P.O. Box 7, Miki-cho Post Office, Miki-cho, Ikenobe 3011-2, Kagawa-ken, 761-0799, Japan

4 Genepioneer Biotechnologies Co. Ltd, Nanjing, 210014, China

[*]Corresponding authors: xhzhang@scib.ac.cn; jaimetex@yahoo.com; magh@scib.ac.cn

Supplementary Table S1. Novel candidate reference genes selected based on RNA-seq data.

Gene name	FPKM root1	FPKM root2	FPKM root3	FPKM heartwood1	FPKM heartwood2	FPKM heartwood3	FPKM heartwood4	FPKM sapwood1	FPKM sapwood2	FPKM sapwood3	FPKM sapwood4	FPKM transition zone1	FPKM transition zone2	FPKM transition zone3	FPKM transition zone4	FPKM leaves	MV	SD	CVs (%)
FAB1A	22.11	23.86	26.51	31.26	26.25	27.18	26.48	29.63	25.69	22.02	25.42	28.84	27.10	24.15	27.06	23.87	26.09	2.54	9.75
UK	2.35	3.31	2.97	2.50	2.67	2.90	2.63	2.82	2.64	2.66	2.32	2.48	2.38	2.89	2.60	2.52	2.67	0.26	9.84
ODD	7.04	9.33	7.93	7.83	6.10	7.82	7.91	7.07	7.60	8.01	8.12	6.47	7.52	7.18	6.67	8.42	7.56	0.80	10.57
Fbp1	4.89	4.53	4.64	3.96	3.97	3.43	3.91	3.76	4.09	3.55	3.73	3.59	3.51	3.73	3.62	3.67	3.91	0.43	11.01
PP2C	8.59	8.41	8.62	8.78	8.16	8.96	8.49	8.36	7.28	6.59	8.53	7.81	8.13	7.77	8.63	11.04	8.38	0.93	11.1
CCS1	5.26	6.15	5.75	4.48	4.66	4.74	5.95	5.42	4.70	4.89	5.76	4.95	4.89	5.21	6.51	5.09	5.28	0.59	11.25
HLMt	9.27	8.26	10.94	8.41	8.62	9.01	7.57	7.31	9.04	9.18	7.61	9.30	8.74	7.85	8.41	10.45	8.75	0.99	11.3
PPR	4.90	4.40	5.12	4.59	5.99	5.85	5.06	5.05	6.11	6.00	5.85	5.48	6.86	5.62	5.81	5.26	5.50	0.64	11.57
Fbp2	9.14	8.40	7.39	8.56	7.91	6.89	8.42	10.70	8.32	8.34	8.01	7.41	8.46	6.70	7.58	8.71	8.18	0.95	11.58
S8	4.28	4.74	3.22	4.16	3.91	3.94	3.89	4.29	4.58	4.18	4.57	4.01	3.66	5.22	3.93	3.57	4.13	0.49	11.81
CSA	16.63	15.18	15.70	17.82	15.83	13.15	12.99	15.36	16.48	14.15	14.62	19.54	14.08	13.04	14.14	14.39	15.19	1.80	11.82
Fbp3	4.15	4.38	5.47	5.47	4.85	4.50	4.68	5.21	4.31	4.72	5.08	6.47	4.87	5.16	4.44	5.51	4.95	0.59	11.95

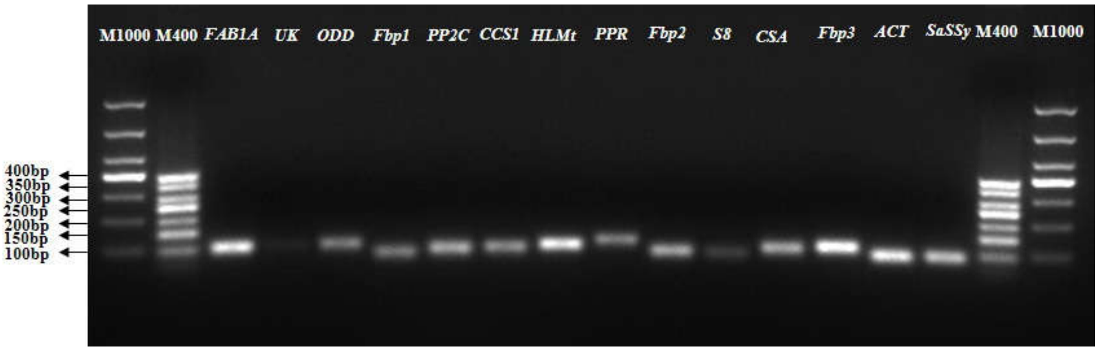

Supplementary figure S1. 2% agarose gel electrophoresis of 13 candidate reference genes

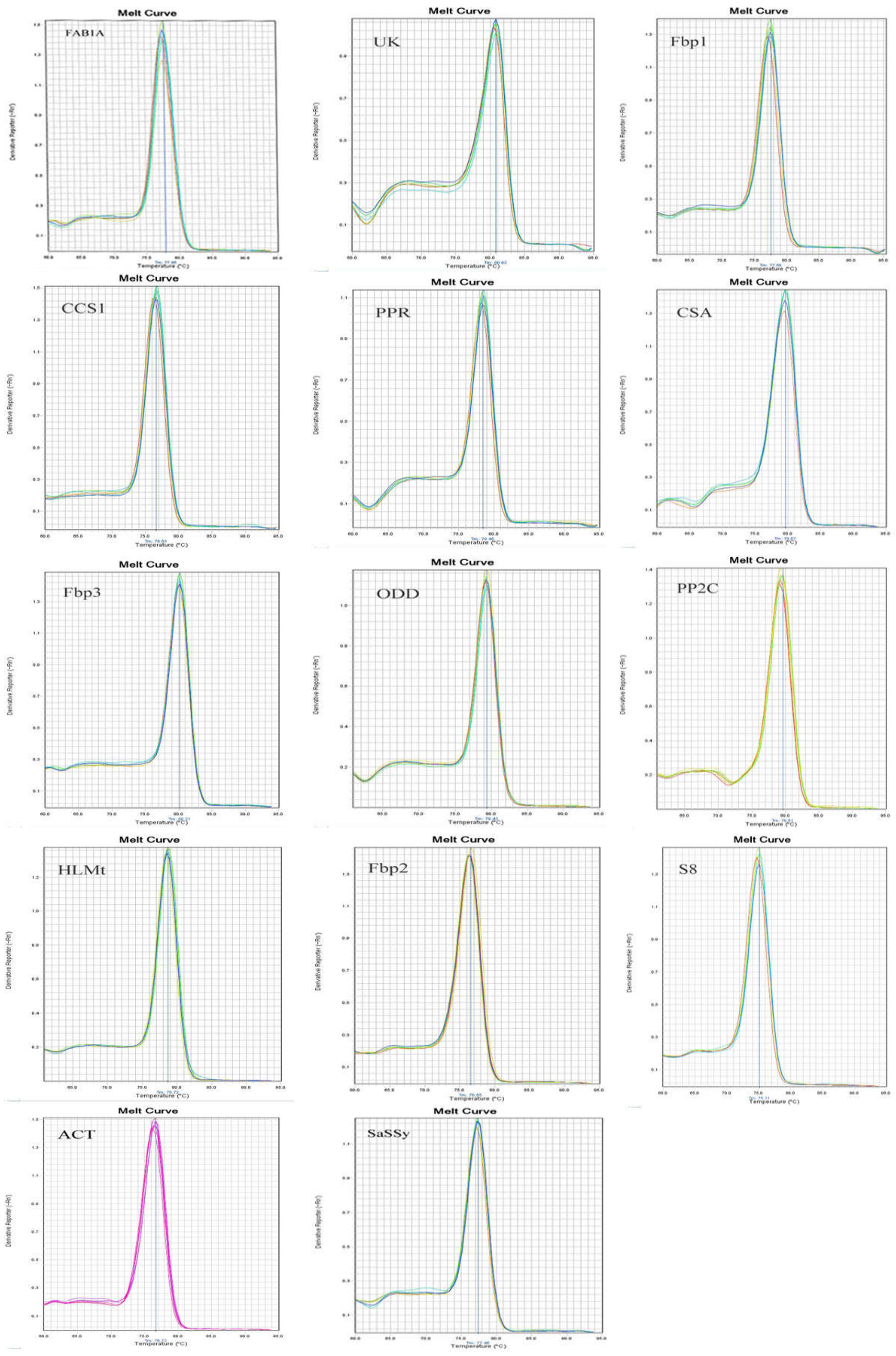

Supplementary figure S2. Melting curve of selected genes
